# Precision of recumbent crown-heel length when using an infantometer

**DOI:** 10.1186/s12887-016-0725-4

**Published:** 2016-11-14

**Authors:** Leila Cheikh Ismail, Fabien A. Puglia, Eric O. Ohuma, Stephen T. Ash, Deborah C. Bishop, Rachel M. Carew, Ayesha Salem Al Dhaheri, Wm. Cameron Chumlea

**Affiliations:** 1 Nuffield Department of Obstetrics & Gynaecology, and Oxford Maternal & Perinatal Health Institute, Green Templeton College, University of Oxford, Women’s Centre, John Radcliffe Hospital, Headley Way, Oxford, OX3 9DU UK; 2 Centre for Statistics in Medicine, Botnar Research Centre, University of Oxford, Windmill road, Oxford, OX3 7LD UK; 3 Ludwig Institute for Cancer Research Ltd, University of Oxford, Nuffield Department of Medicine, Old Road Campus Research Building, Oxford, OX3 7DQ UK; 4 Nutrition and Health Department, College of Food and Agriculture, United Arab Emirates University, Al-Ain, United Arab Emirates; 5 Departments of Community Health and Pediatrics, Lifespan Health Research Center, Boonshoft School of Medicine, Dayton, OH USA; 6 Centre for Tropical Medicine and Global Health, Nuffield Department of Medicine, The Peter Medawar Building, University of Oxford, South Parks Road, Oxford, OX1 3SY UK

**Keywords:** Anthropometry, Growth, Pre-school children

## Abstract

**Background:**

Crown-heel length (CHL) measurement is influenced by technique, training, experience and subject cooperation. We investigated whether extending one or both of an infant’s legs affects the precision of CHL taken using an infantometer. The influence of staff training and infant cooperation were also examined.

**Methods:**

CHL was measured in children (aged 2), infants (aged 1) and newborns, by extending one or both legs. The subject’s level of cooperation was recorded. Mean differences were compared using Student’s *t*-test; intra- and inter-observer variability were assessed using Bland-Altman plots with 95 % limits of agreement. Intra- and inter-observer technical errors of measurement (TEMs) were also calculated.

**Results:**

Measuring CHL in newborns using only one leg resulted in significantly longer measurements. Across all groups, there was less inter-observer variability using both legs; 95 % limits of agreement were lower and TEMs smaller. Larger measurement differences were seen if children were uncooperative.

**Conclusions:**

This study supports measuring CHL with both legs extended. The two-leg technique reduces variability and increases precision by allowing the measurer to control better the position and movements of the infant’s body.

## Background

Anthropometric measurements are a useful clinical toolkit for the assessment of human growth, particularly during infancy. Accurate and precise measurements are essential for reliable monitoring of growth and diagnosis of pathologies in clinical practice and research [1–4]. Extensive training (e.g., locating body landmarks and equipment handling) is necessary to ensure that measurement sites are correctly identified and equipment is used appropriately. Standardisation of technique is important especially if multiple measurers or study sites are involved.

Crown-heel length (CHL) is a reliable and universal indicator of linear growth [5] and nutritional status for infants from birth up to 2 years of age [6]. Techniques for measuring CHL are regularly described in the literature, but assessment of accuracy and precision is often neglected, which may explain the heterogeneity observed across studies. The equipment used also varies from study to study: CHL measurements may be taken using a tape measure, an anthropometric rod, paper-and-pencil, a measuring board or an infantometer [2, 7]. The differences in ease of use and the varying levels of accuracy and precision achieved using these approaches makes it difficult to compare CHL measurements across studies [2, 4, 8–10]. Regardless of the equipment used, CHL is typically measured with both legs extended and the subject’s cooperation is essential for obtaining an accurate measurement [11, 12].

In this paper, we investigate the effects of using one or both legs on the precision of CHL measurement in newborns, infants aged 1 year and children aged 2 years, when using an infantometer. The effects of measurer training and experience, and subject cooperation, are also examined.

## Methods

### Anthropometry

The anthropometry protocol and infantometer (Harpenden, range 300-1100 mm; Chasmors Ltd, London, UK) used in this study were identical to those used in the WHO Multicentre Growth Reference Study (MGRS) [13, 14]. To measure CHL, the infant’s clothes, diaper and any headwear or hair ornaments were removed, and the infant placed supine on the flat base board of the infantometer (for comfort the board was covered with thin cloth or soft paper) with their head held against the head board in the Frankfurt Vertical Plane by an assistant. The measurer gently held and applied pressure to the knees to straighten the legs, while the assistant ensured the hips and shoulders were aligned at right angles to the long axis of the body and the spine was not arched. The measurer then slid the foot board along the base until flat against the soles of the feet, and recorded the measurement from the digital counter (precision ±1.0 mm) to the nearest complete mm. Once recorded, the measurer and assistant switched roles and repeated this procedure, thereby acquiring a pair of independently-obtained CHL measurements. These two values were then compared; if the difference exceeded 7.0 mm (maximum allowable difference, MAD) [13, 14] the measurement was repeated by both observers. In the event that the difference between the second set of measurements also exceeded the MAD, the observers repeated the whole procedure once more. To ensure instrument accuracy, the infantometer was routinely calibrated, twice a week [13].

### Data collection

Four datasets were collected: 1) A set of 194 pairs of lengths from 40 neonates (denoted Newborn A) measured during an anthropometry training session in which nine anthropometrists were trained and standardised against an experienced ‘gold-standard’ anthropometrist [14, 15]. The trainees were split into two groups and each group assigned 20 neonates (1–5 days old). Each trainee measured an infant twice in the presence of an experienced observer and this dataset was used to evaluate intra-observer variability. Each trainee chose at random to measure the CHL of each neonate using either one or both legs. Since there were no significant differences between the two groups (data not shown), the data could be pooled to form this dataset. The other three datasets – 2) Pairs of lengths from 93 neonates (Newborn B), 3) Pairs of lengths from 71 infants at 1 year of age (Infants), and 4) Pairs of lengths from 69 children at 2 years of age (Children) – were all collected as part of routine research assessments, in which pairs of experienced anthropometrists measured CHL once using one leg and once using both, in random order. Sub-samples of the Newborn B, Infants and Children groups were measured a second time to assess intra-observer variability. The behaviour of the Infants and Children groups during measurement was recorded as cooperative or uncooperative, as discerned by the measurers.

### Statistical analysis

CHL measurement precision was assessed by quantifying the intra- and inter-observer variability and the technical error of the measurement (TEM) across the four groups [15, 16], stratified by whether measurements had been taken using the one-leg or two-leg technique. The absolute differences between two measurements performed on the same child by the same observer (intra-observer, “1-leg measurement – 2-leg measurement”) or by two different observers (inter-observer, “Observer 1’s – Observer 2’s measurement”) were plotted against their means and presented as Bland-Altman plots with 95 % limits of agreement [17]. TEMs were calculated for each group as the square root of the sum of the differences between paired measurements divided by twice the number of participants measured [14, 15]. Larger limits of agreement or TEMs were taken to imply greater variability (i.e., less precise measurement). Mean differences between one- and two-leg measurements were compared using t-tests, paired or unpaired as appropriate. All statistical significance was assessed at the 5 % level and all analyses were performed using STATA version 11 (StataCorp, College Station, Texas, USA).

## Results

Neonates in Newborn A were recruited from a regular postnatal ward and were between 1 and 5 days old at measurement; no further demographic details were recorded at the time. However, subjects from the other groups were part of an ongoing study, meaning that supplementary demographic information was recorded. In Newborn B, Infants and Children groups, the mean age (SD) and percentage of male sex were respectively 0.11 (0.39) days and 48.4 %, 12.9 (2.28) months and 52.4 %, and 24.30 (0.94) months and 48.5 %. Means (and standard deviations) of CHL measurements taken using one or two legs in the four analysis groups (Newborn A, Newborn B, Infants and Children) are presented in Table 1. On comparing the mean lengths obtained by the two methods, there was a statistically significant difference in mean CHL for Newborn groups A and B. The mean CHL measured using the one-leg technique was 1.82 cm greater than with the two-leg technique in Newborn A (52.30 cm (2.79) vs. 50.48 cm (2.61), *P* = 0.0001), and 0.09 cm greater in Newborn B (48.38 cm (2.66) vs. 48.29 cm (2.65), *P* = 0.002). There was no significant difference in mean CHL’s between the two measurement techniques for Infants or Children.

**Table 1:** CHL measurements [mean (SD)] and comparison between length measurements obtained extending one or both legs in newborns, infants and children

	Group			
	Newborn A (*N* = 40)	Newborn B (*N* = 93)	Infants (*N* = 82)	Children (*N* = 70)
CHL (cm) measured using:	Mean (SD)	Mean (SD)	Mean (SD)	Mean (SD)
One leg	52.30 (2.79)	48.38 (2.66)	75.84 (3.18)	87.48 (3.48)
Both legs	50.48 (2.61)	48.29 (2.65)	75.82 (3.11)	87.50 (3.50)
*P*-value (*t*-test)	0.0001	0.002	0.56	0.21

**Fig. 1:**
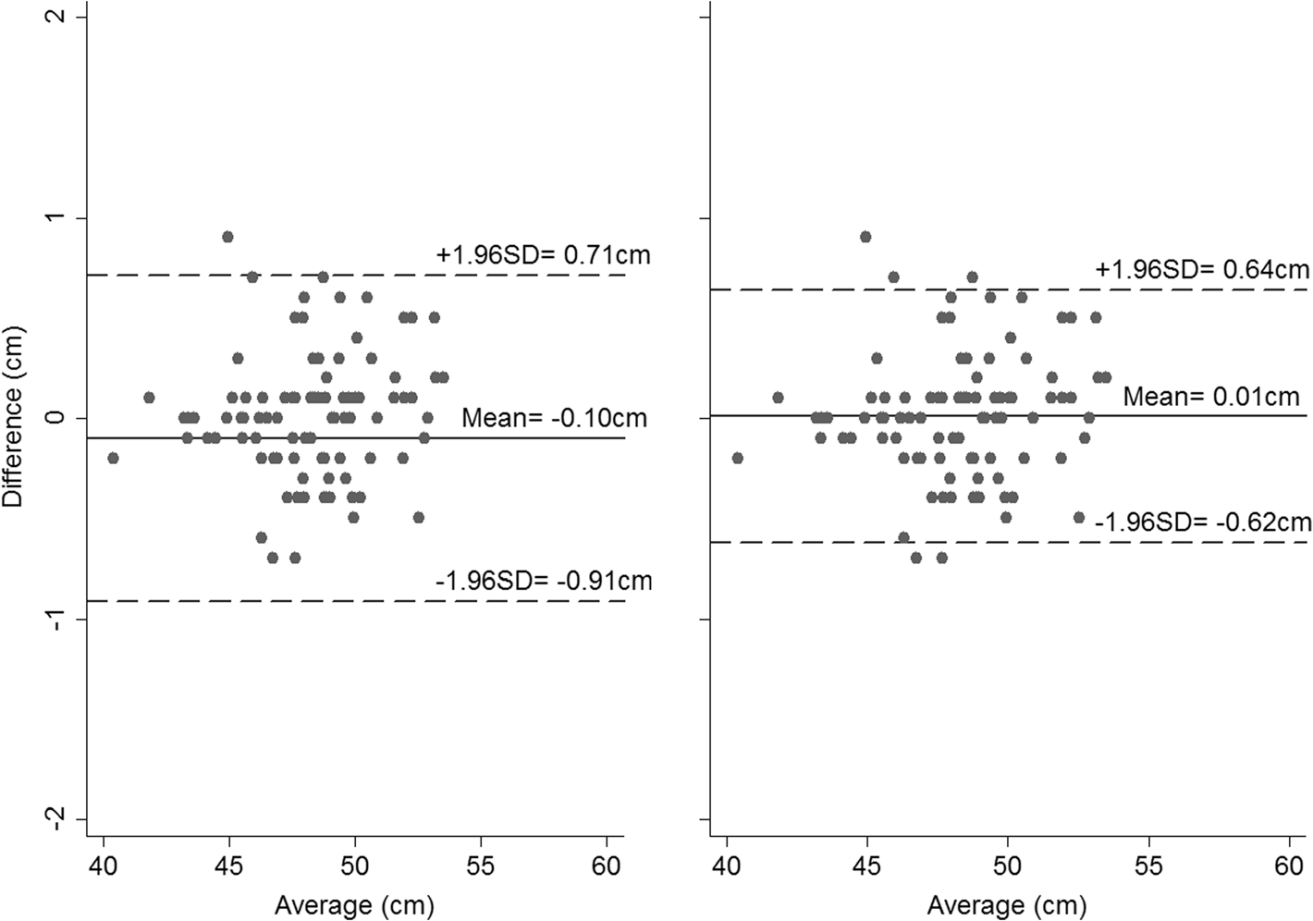
Bland-Altman plots for the inter-observer variability in Newborn B when measured using one leg (*left*) and both legs (*right*) (Differences calculated as Observer 1’s – Observer 2’s measurement)

**Fig. 2:**
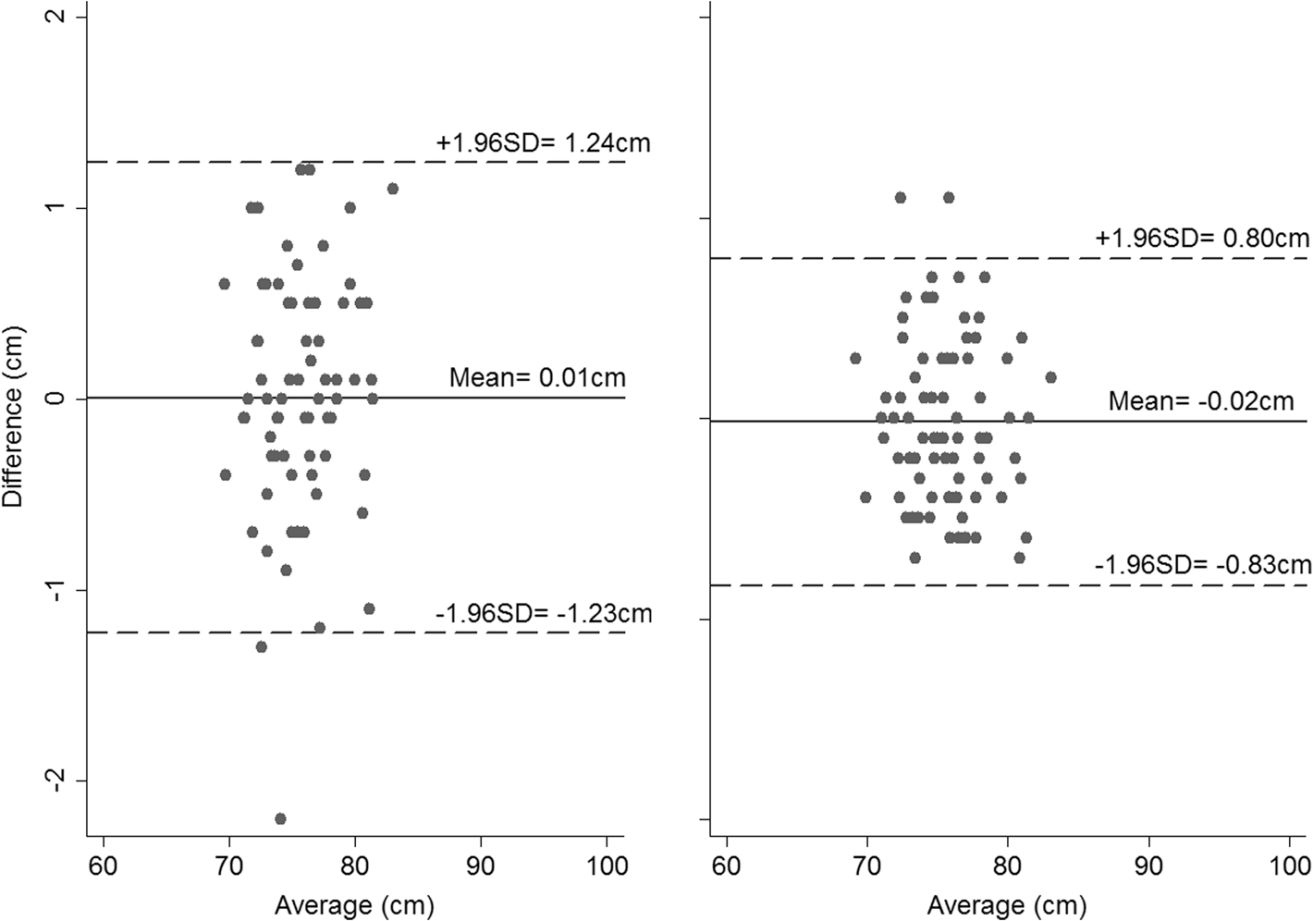
Bland-Altman plots for the inter-observer variability in Infants when measured using one leg (*left*) and both legs (*right*) (Differences calculated as Observer 1’s – Observer 2’s measurement)

**Fig. 3:**
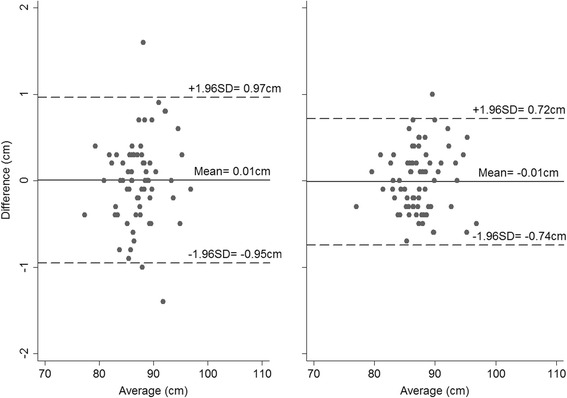
Bland-Altman plots for the inter-observer variability in Children when measured using one leg (*left*) and both legs (*right*) (Differences calculated as Observer 1’s – Observer 2’s measurement)

The mean absolute differences in CHL measurements taken on the same child by two observers are presented in Table 2 for Newborn B, Infants and Children, with corresponding Bland-Altman plots presented in Figs. 1, 2 and 3. The mean absolute differences were similar across the three groups. Overall, the inter-observer variability of CHL measurements was small for each combination of group and technique, but measurements were slightly less variable when using both legs. The 95 % limits of agreement were higher using the one-leg technique (0.81 cm vs. 0.63 cm in Newborn B; 1.23 cm vs. 0.81 cm in Infants, and 0.96 cm vs. 0.73 cm in Children, Figs. 1, 2, and 3 – panel left and right, respectively), and the TEMs were also greater.

**Table 2:** INTER-observer variability, as assessed using Bland-Altman plots and by calculating TEMs in newborns, infants and children

	Group		
	Newborn B	Infants	Children
One leg			
Mean absolute difference (cm)	0.10	0.01	0.01
± 1.96 SD from mean	0.81	1.23	0.96
TEM	0.30	0.44	0.29
Both legs			
Mean absolute difference (cm)	0.01	0.02	0.01
± 1.96 SD from mean	0.63	0.81	0.73
TEM	0.23	0.35	0.27

The mean absolute differences in CHL measurements taken on the same child by the same observer are presented in Table 3 for Newborn A, Newborn B and pooled data from the Infants and Children groups (since only a small number of 1- and 2-year-old subjects were measured twice by the same observer). For the Newborn B and pooled Infants/Children groups, the mean absolute differences were similar, while the intra-observer variability of CHL measurements was small for each combination of group and technique, and the measurements slightly more variable when using one leg. The 95 % limits of agreement were higher using the one-leg technique (1.21 cm vs. 0.68 cm in Newborn B, and 0.75 cm vs. 0.70 cm in pooled Infants/Children), and the TEMs were slightly greater. However, for Newborn A, the mean absolute difference was smaller when using the one-leg technique, and although the 95 % limits of agreement were similar for the two techniques, the TEM was smaller when measuring CHL with one leg.

**Table 3:** INTRA-observer variability, as assessed using Bland-Altman plots and by calculating TEMs in newborns, infants and children

	Group		
	Newborn A	Newborn B	Infants/Children (pooled)
One leg			
Mean absolute difference (cm)	0.08	0.15	0.13
± 1.96 SD from mean	1.12	1.21	0.75
TEM	0.35	0.30	0.27
Both legs			
Mean absolute difference (cm)	0.28	0.11	0.13
± 1.96 SD from mean	1.10	0.68	0.70
TEM	0.42	0.27	0.25

The impact of subject cooperation on inter-observer variability of CHL measurements is presented for pooled Infant and Children group data in Table 4. The mean absolute difference was slightly larger in uncooperative than in cooperative subjects for both measuring techniques (0.14 cm vs. 0.03 cm when measuring with one leg, and 0.07 cm vs. 0.05 cm with two). Although the 95 % limits of agreement were similar for both groups measured using one leg (1.11 cm vs. 1.10 cm), uncooperative children measured using two legs showed more variable measurements than their cooperative counterparts measured using the same technique (0.87 cm vs. 0.73 cm).

**Table 4:** INTER-observer variability, as assessed using Bland-Altman plots, by infant behaviour, in infants and children

	Infant behaviour^a^	
	Cooperative	Uncooperative
One leg	*N* = 109	*N* = 32
Mean absolute difference (cm)	0.03	0.14
± 1.96 SD from mean	1.10	1.11
Both legs	*N* = 109	*N* = 40
Mean absolute difference (cm)	0.05	0.07
± 1.96 SD from mean	0.73	0.87

^a^Data from Infants and Children groups pooled together

## Discussion

Newborns, infants and small children are difficult to measure because of their very small size and inability to follow verbal instructions or control their body movements, especially if they are temperamental. In addition, a crying infant is unsettling to both staff and parents [5], which can cause the measurer to hurry. All these factors create a set of problems which can result in errors that are large compared to the small measurement values [7, 13, 14]. The present findings shed some light on the influence of technical variations (extending one or both legs), training (experienced vs. trainee staff) and subject cooperation on CHL precision.

Our findings indicate that measuring CHL in newborns using only one leg resulted in significantly greater length measurements than when measuring with both legs (1.82 cm and 0.09 cm longer when taken by trainee anthropometrists in Newborn A, and experienced measurers in Newborn B, respectively). This is consistent with the findings of other researchers who have previously reported a difference between the two techniques [18]. There was less variability in CHL measurements between different measurers when extending both legs rather than one, regardless of age group or subject cooperation. Moreover, when using the two-leg technique, experienced anthropometrists showed less variability when re-measuring the same subject than when using only one leg. However, trainee anthropometrists encountered more difficulties when using the two-leg technique, demonstrating slightly larger differences in their repeated measurements and greater TEM when measuring with two legs. This proves that using both legs to measure CHL is the more difficult of the two techniques for a novice; however, once mastered, this approach consistently produces precise results. As expected, when infants or children are uncooperative, slightly larger differences are seen in measurements obtained by separate observers. Although measurements taken using the two-leg technique become more variable when a child is uncooperative, it should be noted that this method is still more precise than if one leg is used. Therefore, extending both legs when measuring a subject’s length helps to position the lower half of their body correctly. First, it prevents the subject from resisting by having both legs immobilised by the observer. Secondly, the hip joints, if both extended, are more likely to be in the same position and can easily be adjusted to be perpendicular to the long axis of the body.

Of the factors affecting CHL measurement, the choice of measuring equipment is critical and should be made based on expected accuracy and precision, whilst taking into account where the measurements will be taken and by how many people. The present study used the Harpenden infantometer, which is recognised to be accurate and precise but expensive. Measurers’ training, experience, reliability, fitness and mood also play important roles in determining the final measurement value. Although the latter two may be considered subjective, hard to quantify and variable between and within days, the other factors can – and certainly should – be incorporated into the study design. Training should address technical issues such as locating correct landmarks and body position (which has previously been reported as the greatest source of measurement error [19]). As demonstrated, small variations in protocol – e.g., using one or both legs – can impact on data quality, especially when interacting with other factors. When this study was conducted, our experienced measurers were already familiar with the equipment and the techniques, had undergone rigorous standardisation, and had each previously taken measurements in this way on many newborns, infants and children [15]. In contrast, most trainees are novices at anthropometry, but their performance improves with further training and standardisation [4, 15, 20, 21]. Although advising on how much training is required to achieve competency is behind the scope of this paper, in our study all the measurers received the same training according to the WHO MGRS and the INTERGROWTH-21^st^ Project training recommendations [14, 15]. This consisted of a short theoretical session describing the technique with the opportunity to practice under supervision on a small number of subjects, followed by a formal ‘standardisation’ session on 20 subjects with comparison to an expert measurer. Results from standardisation indicated whether a measurer required further training or practice.

When measuring CHL in neonates a great deal of care should be taken, bearing in mind their fragility [18]. However, neonates are largely unaffected by the presence of the measurers and can be easily soothed, whereas infants and children are more aware of their surroundings and can be wary in an unfamiliar environment and thus uncooperative.

The present study shows that technical choices, such as one vs. two legs, are important and that training is paramount. Measuring neonatal CHL with one leg is easier than with both legs as demonstrated here but results in greater variability and significantly greater CHL values. Measuring length using both legs allows the observer to control better the position of the subject’s body on the infantometer and decrease the effect of his/her uncooperativeness on the measurement. The use of both legs when measuring CHL should be encouraged but, if not possible, one leg can be held in place to take the measurement. However, when this is necessary, the change in technique should be noted, as use of this alternative method can affect data quality. The same applies whenever an older child is uncooperative.

## Conclusion

When measuring CHL in children under 2 years of age, whether in a clinical or research context, attention to technique and training improve data quality. From a technical point of view, measuring CHL with both legs extended should be encouraged as it allows for better control of the position and movements of the subject’s body and, therefore, reduces variability and increases precision.

## Abbreviations

CHL: Crown-heel length
MAD: Maximum allowable difference
MGRS: Multicentre Growth Reference Study
TEM: Technical error of measurement
